# Functional Diffusion Tensor Imaging: Measuring Task-Related Fractional Anisotropy Changes in the Human Brain along White Matter Tracts

**DOI:** 10.1371/journal.pone.0003631

**Published:** 2008-11-04

**Authors:** René C. W. Mandl, Hugo G. Schnack, Marcel P. Zwiers, Arjen van der Schaaf, René S. Kahn, Hilleke E. Hulshoff Pol

**Affiliations:** 1 Rudolf Magnus Institute of Neuroscience, University Medical Center Utrecht, Utrecht, The Netherlands; 2 Donders Institute for Brain, Cognition and Behaviour, Centre for Cognitive Neuroimaging, Radboud University Nijmegen, Nijmegen,The Netherlands; Lund University, Sweden

## Abstract

**Background:**

Functional neural networks in the human brain can be studied from correlations between activated gray matter regions measured with fMRI. However, while providing important information on gray matter activation, no information is gathered on the co-activity along white matter tracts in neural networks.

**Methodology/Principal Findings:**

We report on a functional diffusion tensor imaging (fDTI) method that measures task-related changes in fractional anisotropy (FA) along white matter tracts. We hypothesize that these fractional anisotropy changes relate to morphological changes of glial cells induced by axonal activity although the exact physiological underpinnings of the measured FA changes remain to be elucidated. As expected, these changes are very small as compared to the physiological noise and a reliable detection of the signal change would require a large number of measurements. However, a substantial increase in signal-to-noise ratio was achieved by pooling the signal over the complete fiber tract. Adopting such a tract-based statistics enabled us to measure the signal within a practically feasible time period. Activation in the sensory thalamocortical tract and optic radiation in eight healthy human subjects was found during tactile and visual stimulation, respectively.

**Conclusions/Significance:**

The results of our experiments indicate that these FA changes may serve as a functional contrast mechanism for white matter. This noninvasive fDTI method may provide a new approach to study functional neural networks in the human brain.

## Introduction

Neurobehavioral functions depend on a dynamic flow of information between various gray matter brain regions which are interconnected via white matter pathways [1], [2]. Activation of the human brain's gray matter regions have been extensively studied with neuroimaging techniques such as functional magnetic resonance imaging (fMRI), positron emission tomography (PET), single photon emission computed tomography (SPECT), electro-encephalography (EEG) and magneto-encephalography (MEG). However, these techniques do not provide information on the white matter pathways and their corresponding activity. Imaging techniques such as DTI [3], [4] in combination with fiber tractography [5]–[7] allow us to non-invasively study the anatomy of these pathways-but not their activity. In this paper we present a functional diffusion tensor imaging (fDTI) method that is able to detect task-related changes in FA that may represent the activity of white matter tracts. Directly testing the white matter tracts for activation would provide us with a unique opportunity to study the connections of neural networks that become active during various cognitive functions. In addition, it may allow the study of dysfunctional neural networks in patients with neurological and psychiatric diseases, such as schizophrenia in which structural and functional brain abnormalities have been found [8]–[10].

In DTI the diffusion profile of water molecules is measured. In white matter fibers, which are formed by large numbers of heavily myelinated axons running in parallel, the diffusion is more hindered in the radial directions (i.e. perpendicular to the fibers' principal direction) than in the direction parallel to the fibers. As a result, the diffusion profile in white matter is elongated (cigar shaped), pointing in the direction of the fibers. In DTI the diffusion profile is represented by a positive definite tensor of which the major eigenvector with eigenvalue λ_1_ represents the direction parallel to the fibers and both minor eigenvectors with eigenvalues λ_3_ and λ_3_ represent the radial directions.

The fractional anisotropy [11] (FA) defined by:
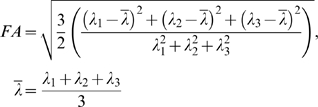
is a relative measure which describes the variance between the levels of diffusion measured in the different directions. It is therefore not sensitive to fluctuations in the diffusion unweighted (b = 0 s/mm^2^) image, which serves as normalization factor in the eigenvalue computation. This in contrast to other measures such as radial diffusivity defined by (λ_2_+λ_3_)/2, parallel diffusivity defined by λ_1_, or mean diffusivity defined by (λ_1_+λ_2_+λ_3_)/3, which are sensitive to intensity fluctuations in the diffusion unweighted image, for instance due to task-related onset changes in T_2_^*^ (the basis of the fMRI BOLD contrast). The FA is therefore well suited to study activation-related changes in white matter as it is insensitive to possible task-related T_2_^*^ changes in neighboring gray matter.

fDTI is based on the assumption that changes in FA are a sign of local fiber activity. We hypothesize that morphological changes of glial cells (in particular oligodendrocytes) lead to shape changes of the extra-cellular space (ECS) [12]–[14] and, in turn, lead to a measurable increase in FA. Indeed, changes in the diffusion profile due to changes in the ECS in white matter have been shown in vitro using diffusion weighted imaging in the rat optic nerve [15]. An earlier study [16] reported that electrical stimulation induced significant changes in the diffusion properties of brain tissue in rats. Using intrinsic optical signal (IOS) measurements [17] slowly varying activity-related signal changes were measured in the rat optical nerve and were attributed to glial cell swelling. However, a study using the real-time tetramethylammonium (TMA^+^) iontophoretic method in combination with IOS measurements [18] showed that the concentration of TMA^+^ in the extra cellular space (ECS) did not change although similar changes in the IOS signal were measured. Therefore the authors concluded that it was unlikely that glial cell swelling was the primary mechanism for these IOS changes and they suggested that a more plausible explanation may be found in morphological changes of glial cells. A recent study [19] showed that increased levels of potassium lead to changes in both IOS values and MRI proton density measurements for gray and subcortical white matter in rats, suggesting that activity-related changes in the ECS of gray matter as well as white matter can be measured using MRI. The authors therefore conclude that cell swelling arising from neuronal depolarization is detectable with fMRI-like acquisitions.

With respect to the measurement of activation in gray matter there is an ongoing discussion whether diffusion-weighted MRI provides a more direct way to measure activation than functional MRI methods that are based on vascular responses such as BOLD contrasted fMRI. Le Bihan and colleagues [20] hypothesized that for functional imaging of gray matter diffusion-sensitized images can be used to measure neuronal activity-related cell swelling. This would provide a more direct way to measure neuronal activation than standard BOLD contrasted fMRI does. The results of their experiments not only suggested that the measured diffusion-sensitized MRI signal changes could be linked to activity-related cell swelling but also that the delay between stimulus onset and measured signal change was notably smaller for the diffusion-sensitized MRI signal than the delay between the stimulus onset and the BOLD fMRI signal. However, another study [21], which compared signal changes induced by neuronal activation and signal changes due to hypercapnia, was not able to replicate this difference in delays. Moreover, the results of that study suggested that even with strong diffusion weighting vascular effects could not be ruled out as a possible source for the measured task-related MRI signal change.

With respect to the measurement of activity in white matter, several different physiological processes-other than morphological changes of glial cells-that are part of (or accompany) fiber activity could, in theory, alter the diffusion profile and therefore the measured FA-value as well. For instance, the changes in FA-value may also have been altered by local changes in capillary blood flow [3], in capillary blood volume [22] or by activity-related anisotropic magnetic susceptibility variations [23]. These other possible explanations for the observed task-related changes in FA should be considered and will be addressed in the discussion section.

**Figure 1:**
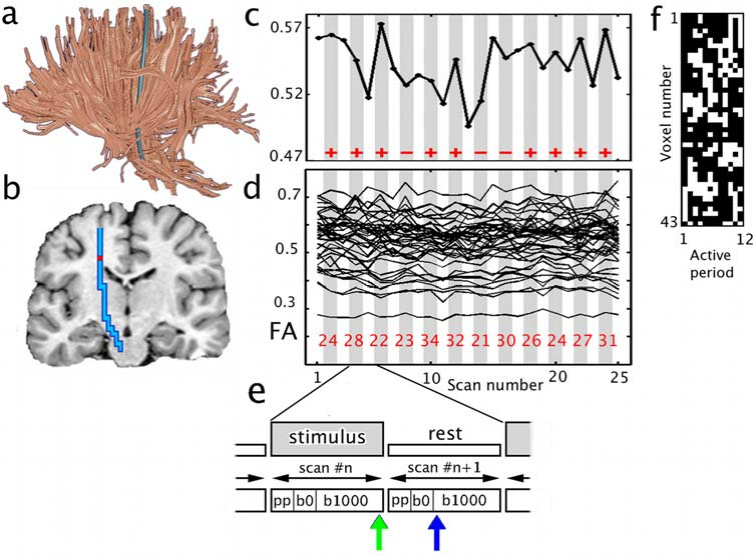
Overview of the fDTI analysis. (a) The fDTI method starts with the reconstruction of anatomical tracts in the brain without any region specific selection criteria using conventional fiber tracking. All the tracts (here the total count for the whole white matter was 20,193 tracts) are then tested individually for activation. (b) The blue colored tract is an example tract used to explain the test for activation. (c) In the fDTI method the FA-values during rest are compared to the FA-values during the task. These changes in FA-value are encoded per voxel using a series of ‘+’s and ‘−’s. If the FA-value of an active period (gray column) was higher than the average FA-value of its temporal neighbors (both resting periods, shown in a white column) then a ‘+’ was assigned, and otherwise a ‘−’, here shown for the individual red voxel. Note that the test for activation is done over the complete tract, and not at the level of individual voxels, as we assumed that during a task the whole tract is active. In this way the statistical power is increased allowing subtle FA-changes to be detected. (d) The FA-values measured for all 43 voxels that are part of the tract that is tested. The numbers shown for each active period at the lower part of the graph (in red) depict the number of ‘+’s encoded over all 43 voxels of the tested tract. The total number of ‘+’s found was 322 from a total of 516 (12×43) signs. The sign-test yielded p = 0.000000001 for the tested tract and remained significant at the 0.05 level even after Bonferroni-correction for the total number of tracts tested (Bonferroni corrected threshold is 0.05/20193 = 0.000002). (e) In this timing plot of a stimulus and resting epoch pp stands for preparation phase (12 s), b0 denotes the time period in which the b = 0 s/mm2 volume is acquired (12 s), and b1000 denotes the time period in which the six b = 1000 s/mm2 volumes are acquired (36 s). The green and blue arrows denote where (according to the results of the time course experiment) the maximum signal change is expected for the tactile and visual stimulus, respectively. (f) shows the matrix with all the signs of the active tract. The ‘+’s are represented by black and white denotes the ‘−’s. This sign matrix shows that the positive correlation of the tract with the task is likely to represent signal changes over large parts of the tract over a considerable time period.

Normal activity-induced changes in the diffusion profile of white matter are expected to be very small as compared to the physiological noise [24] and a reliable detection of the signal change would require a large number of measurements. But if it is assumed that activity-related FA-signal changes extends the whole tract, then a substantial increase in signal-to-noise ratio can be achieved by pooling the signal over the entire tract. It is the adoption of a tract-based statistics -rather than a voxel-based statistics- that enables us to measure the signal within a practically feasible time period. The principle of the fDTI method is outlined in Figure 1.

To assess the validity of the fDTI method, eight healthy right-handed subjects participated in both tactile and visual fDTI experiments. Afterwards, three other subjects participated in a second, time course experiment. The second experiment provided more specific information on when the maximum of the measured signal change is found for the different types of stimuli. Throughout the rest of this paper we will refer to the first experiment as the fDTI experiment and to the second experiment as the time course experiment. These experiments were approved by the medical ethical committee for human subjects of the University Medical Center Utrecht, The Netherlands, and all subjects provided written informed consent prior to participation.

## Materials and Methods

### fDTI experiment

In the tactile experiment the subjects were instructed to keep their eyes closed for the duration of the whole experiment. During the active condition, the palm and fingers of the subject's right hand were brushed in a random fashion by an investigator. In the visual experiment the subjects were instructed to look at a red fixation cross that was projected on the center of a screen visible from inside the scanner at all times. During the active condition a black and white checkerboard was shown that alternated at a frequency of 8 Hertz. During the rest condition only the red fixation cross was visible. In both fDTI experiments the same active versus rest paradigm was used and the scan parameter settings were identical. These two fDTI experiments were selected for their expected lack of overlap in activated tracts, which allowed us to study both the method's specificity and sensitivity. Moreover, a subject's response was not required in either of the tasks reducing the chance of task-related motion artifacts.

For each fDTI experiment a separate anatomy scan, a conventional high-resolution DTI scan and an fDTI scan were acquired (see MRI scan acquisition). The subjects left the scanner room to rest between the two experiments for at least 15 minutes. For one subject the results of the visual task were excluded because of visibility problems of the checkerboard reported afterwards. The conventional DTI-scan was used for reconstruction of the tracts (Figure 1a) using the FACT-algorithm [25]. The anatomy scan was used for inter-subject registration and visualization of the results. We did not use cardiac gating as it would lengthen the acquisition time considerably. Identical timing parameters for the visual task and the tactile task were used to make direct comparison possible between the results of the tactile and visual task.

#### MRI scan acquisition

For the fDTI experiment the following scans were acquired on a Philips Achieva 1.5 Tesla whole-body MR scanner (Intera Achieva, Philips, Best, The Netherlands). First, a T_1_-weighted whole brain scan was acquired for anatomical reference. Next, a conventional transverse single shot spin-echo, echo planar imaging (SS-EPI) DTI scan was acquired for reconstruction of the white matter tracts in the whole brain (acquisition matrix = 128×96; FOV = 240 mm; 60 slices; slice-thickness = 2.5 mm; no gap; TE = 86 ms; TR = 10 000 ms; parallel imaging SENSE factor = 2; 32 different diffusion gradient directions with b-factor = 1000 s/mm^2^; scan duration = 354 s). The functional time series of DTI scans (the fDTI set) were acquired during the execution of an alternating sequence of a neurobehavioral task and a resting condition. A total of 29 transverse SS-EPI DTI scans (acquisition matrix = 64×64; FOV = 256 mm; 40 slices; slice-thickness = 4 mm; no gap; TE = 74 ms; TR = 6000 ms; parallel imaging SENSE factor = 2.5; 1 scan without diffusion gradients and 6 non-collinear diffusion gradient directions with b-factor = 1000 s/mm^2^; scan duration per DTI scan = 60 s) were collected (15 during the resting condition and 14 during the active condition; beginning and ending with a resting condition).

These DTI scans started with a calibration period of 12 seconds. For a stimulus period the stimulus started at the beginning of the calibration to eliminate possible onset effects of a BOLD related signal during the acquisition of the data (BOLD signal has a time-to-peak that is typically below 10 seconds). Next, two diffusion unweighted volumes (which are averaged) with a total duration of 12 seconds were acquired, followed by 6 diffusion weighted volumes (36 seconds) using the following diffusion gradient scheme: (Gx, Gy, Gz) = (1, 0, 0), (0, 1, 0), (0, 0, 1), (−½√2, 0, −½√2), (½√2, ½√2, 0), (0, ½√2, ½√2), where the x-, y-, and z-axis correspond to the patient's right-left, anterior-posterior, and feet-head direction, respectively. The first 4 DTI scans were disregarded to eliminate possible onset effects (for example, due to heating of the scanner gradients) leaving 13 rest scans and 12 active scans.

#### Post processing

The diffusion unweighted scan of the first fDTI image was used to compute the rigid body transformation that aligns the first fDTI image with the conventional DTI image. Because the application of parallel imaging substantially reduces susceptibility artifacts (e.g. nonlinear spatial deformations) we assumed that rigid body transformations could be used in this registration step. All other images in the fDTI set were then registered (using cross correlation) towards the first fDTI image. In this step each diffusion weighted scan in the other series was registered to its corresponding diffusion-weighted scan of the first fDTI image. Subsequently the FA was computed for each separate voxel of each image in the fDTI set. The conventional DTI image was used to reconstruct the tracts for the whole brain with an in house implementation of the FACT algorithm [25]. Parameter settings: minimum FA>0.2, maximum angle between current major eigenvector and previous major eigenvector <26 degrees, average maximum angle between current major eigenvector and major eigenvectors of neighboring voxels (R-value) <37 degrees, minimum tract length 50 mm, number of tract starting points per voxel = 8.

#### Statistical analysis

Considering that the FA-signal change extends over the whole tract we postulated that a switch to statistical testing at the level of complete tracts instead of single voxels, yields the necessary increase in statistical power (see the legend of Figure 1 for a detailed explanation). However, white matter voxels that are part of an *active* tract may all have different baseline FA-values and their (non Gaussian) distributions are likely to differ as well. Because of these differences in baseline FA-values the comparison between active and rest FA-values should be done *per voxel* in order to keep the within variation as low as possible. Also, because of the expected different distributions, the conservative nonparametric sign-test [26] was used as a test-statistic because this test does not require normally distributed data. For each separate voxel that was part of the tested tract, the FA-values measured during the active conditions were compared to rest FA-values and were encoded by a series of ‘+’ and ‘−’ signs as follows. An active condition was assigned a ‘+’ if its FA-value was higher than the average of the FA-values of its two temporal neighbors (the rest conditions just before and just after the rest conditions), otherwise a ‘−’ was assigned. To test if a complete tract was active, the sign-test was applied to the set of ‘+’ and ‘−’ signs combined for all voxels that were part of the tract. Thus, if the number of voxels which are part of the tract that is tested for activation is *n* and the number of signs per voxel is *m*, then the sign-test is applied to a set of *n*×*m* signs. If, by using the sign-test with a significance threshold of p<0.05, the number of ‘+’s found differed significantly from what was expected according to the binomial distribution with equal probabilities for ‘+’ and ‘−’, the tract was considered active. When all tracts were tested for activity in this manner, the significance level of 0.05 should be properly adjusted to correct for multiple testing. In this study the Bonferroni correction was used.

#### Multi-subject averaging

To compare the results of the tactile and the visual fDTI experiment at a group level the individual results were placed in a common space as follows. For each subject a binary map of the complete set of voxels that coincides with the active tracts found was placed in one common space using the affine transformation that registers the subject's anatomy scan with the Montreal Neurological Institute MNI-305 template. The affine transformation was computed using the ANIMAL algorithm [27] and the resampling of the binary maps was performed using linear interpolation. Each of the transformed maps was then blurred with a 3-dimensional Gaussian kernel with a full width at half maximum of 7 mm (to partially overcome spatial inter-subject variability) followed by a threshold at a value of 0.1 yielding a second binary map. The result of this procedure is that the binary voxel representation of an active tract in the second binary map is a dilated version of the binary voxel representation of an active tract in the first binary map. Finally these binary maps of the subjects were accumulated and overlaid on the subjects' average anatomy. Thus the value of a (colored) voxel represents the number of subjects for which an active tract can be associated with that voxel.

#### Average percent FA-signal change

For each subject the per voxel average percent FA-signal change ( (FA_task_−FA_rest_)/FA_rest_)×100% was computed for the FA time series of the voxels that are part of active tracts. The per voxel average percent FA-signal change values were computed and were averaged over all active voxels of all subjects yielding an overall average percent FA-signal change. Likewise, the per voxel average percent signal change for the same set of active voxels was computed for the parallel diffusivity, the radial diffusivity and the mean diffusivity.

#### Estimation of the contribution of blood volume changes

To obtain a coarse estimate of the maximum contribution of possible task-related blood volume changes to the measured signal we used a two-compartment model [28] consisting of an intra- and extravascular component. The signal *S_r_* during rest condition was modeled by:
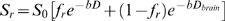
and the signal *S_a_* during active condition was modeled by:
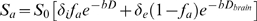
where *S_0_* is the baseline signal without diffusion weighting, *f_r_* and *f_a_* are the volume fractions of the intravascular component during rest and activation, *D* is the apparent diffusion coefficient (ADC) of the vessel, *D_brain_* is the ADC of brain tissue, and *δ_i_* is a composite coefficient for intravascular and *δ_e_* for extravascular contributions to the signal changes (reflecting T_2_ and potentially apparent T_1_ activation related changes). The following parameter settings were used: *f_r_* = 0.01 (for white matter, the fraction of the microvascular tissue is in the order of 1% [29], [30]); *f_a_* = 0.015 (i.e. 50% change in volume fraction with activation, based on [31]); b-factor = 1000 s/mm^2^; *D* = 1×10^−3^ mm^2^/s (capillaries). Assuming that the extravascular component contains white matter two different ADC values ranges for brain tissue were used *D_brain_* = [2.46×10^−4^ mm^2^/s, 5.23×10^−4^ mm^2^/s] (radial) and [1.51×10^−3^ mm^2^/s, 1.83×10^−3^ mm^2^/s] (parallel) [32]. The composite coefficients were set to *δ_i_* = 1.2 (here the estimate for gray is matter used) and *δ_e_* = 1 (no extravascular contribution). The range of the relative signal change (*S_a_*−*S_r_*)/*S_r_* in the radial direction is [−1.2×10^−3^, −3.50×10^−5^] and the parallel direction range is [8.27×10^−3^, 1.32×10^−2^] which correspond to changes in ADC in the range [1.24×10^−6^ mm^2^/s, 3.50×10^−8^ mm^2^/s] (radial diffusion) and [−8.23×10^−6^ mm^2^/s, −1.31×10^−5^ mm^2^/s] (parallel diffusion), respectively. Thus the maximum signal changes induced by blood volume are therefore estimated for radial diffusivity between 0.50% and 0.01% and for parallel diffusivity between −0.54% and −0.72%.

### Time course experiment

Considering the results of the fDTI experiment the signal contrast between stimulus and rest conditions was expected to be maximal when the diffusion gradient direction is perpendicular to the tract. Because the “tracts of interest” were known beforehand (i.e. the sensory thalamocortical tract and the optic radiation), for the time course experiment, a single diffusion gradient direction was selected that is perpendicular to the tract of interest. By selecting only one diffusion gradient direction instead of six (necessary to compute the FA) the temporal resolution was increased to 2.6 seconds.

#### MRI scan acquisition

The scans for the time course experiment were acquired on a Philips Achieva 3 Tesla whole-body MR scanner (Intera Achieva, Philips, Best, The Netherlands). For the time course experiment with the tactile stimulus, a transverse SS-EPI diffusion weighted scan with 80 diffusion weighted volumes was acquired with the same diffusion gradient applied in the subject's left-right direction for each of these diffusion weighted volumes (acquisition matrix = 80×80; FOV = 200×200 mm; 28 slices; slice thickness 7.5 mm; no gap; TR = 2852 ms; TE = 68 ms; diffusion gradient b-factor = 1000 s/mm^2^; parallel imaging SENSE factor = 3; total scan duration 239 s). Note that for this experiment no diffusion unweighted volumes were acquired because this would decrease the temporal resolution. The scan was repeated 5 times and anisotropic voxels aligned with the tracts of interest were used to reduce partial voluming to increase the signal to noise ratio. The same tactile stimulus was used as in the tactile fDTI experiment. The stimulus started at the 4^th^ scan and ended after the 26^th^ scan (stimulus duration 60 s). The scan parameters for the time course experiment with the visual stimulus were identical to the scan parameters for the time course experiment with the tactile stimulus except that the slice direction was set to coronal and that the diffusion gradient was applied in the subject's upper-left bottom-right direction (the usage of a combined diffusion gradient in the visual task was necessary to keep the TE of the visual task identical to the TE of the tactile task). The stimulus used was the same stimulus as used in the visual fDTI experiment and the stimulus started at the 4^th^ scan and ended after the 26^th^ scan. The conventional DTI scan used in the time course experiment was identical to the one described in the fDTI experiment.

#### Post processing

For the analysis of the scans of the time course experiment with tactile stimulation all voxels that were part of the tracts connected to the left sensory thalamocortical tract were selected. From these voxels, 25 voxels with the highest MRI signal (averaged over all scans) were selected being the “voxels of interest” for these voxels a task-related signal change was expected. These voxels were selected in this way because a high MRI signal is expected in white matter when the direction of the diffusion gradient is perpendicular to the tract's main direction. The MRI signals of these voxels of interest were normalized by dividing their values by the (per voxel) medians. In contrast to the fDTI experiment, only diffusion weighted (b = 1000 s/mm^2^) data were acquired for the time course experiment, and thus no normalization with respect to the diffusion unweighted signal (b = 0 s/mm^2^) was possible, making these data susceptible to global signal variation. To correct for the influence of global signal variation, the MRI signal values of the voxels of interest were covariated with the MRI signal values of voxels that had similar signal intensity characteristics but were not expected to show a task-related signal change: the “background voxels”. These background voxels were the 25 voxels with the highest average MRI signal value selected from tracts that were not part of the sensory thalamocortical tract. The 15 measurements (3 subjects; 5 measurements per subject) were averaged and smoothed using a Gaussian kernel with sigma = 14 s. In a similar fashion the analysis of the scans for the time course experiment with the visual stimulus was carried out. The only difference was that the 25 voxels of interest were selected from the optic radiation and that the background voxels were selected from tracts other than the optic radiation.

## Results

**Figure 2:**
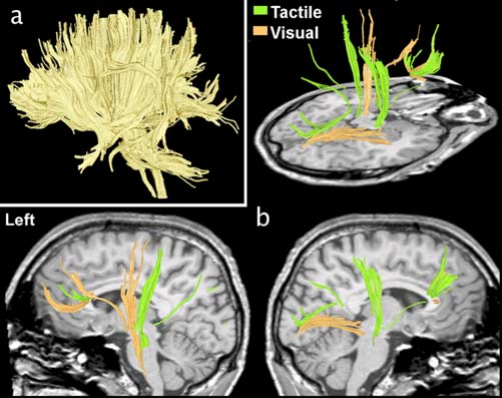
Individual fDTI results for subject 5. (a) The reconstructed tracts that were tested for activity using the fDTI method. (b) Tracts that were found positively active during the visual task (orange) and the tactile task (green). The lower images show the tracts found for the left and the right hemisphere. During the tactile task, positively correlated activation was found predominantly contra-laterally for thalamocortical tracts running to the primary sensory cortical area. Positively correlated activation during the visual task was found amongst others for tracts that are part of the optic radiation.

**Figure 3:**
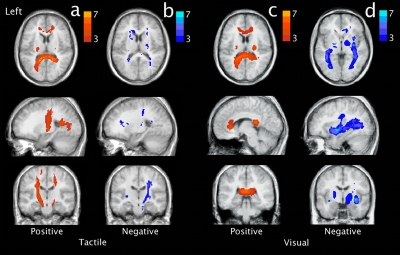
Accumulated group fDTI results for the tactile and visual task. For both tasks the accumulated group fDTI results were computed and overlaid on the subjects' average anatomy. The color of the voxel represents the number of subjects for which active tracts are found at that position. Red voxels denote positively correlated activation (a,c) while blue voxels denote negatively correlated activation (b, d). Note that for the visual task the slices for the positively correlated activation (c) and negatively correlated activation (d) were taken at different positions. The results for the positively correlated activation showed that the majority of the positively correlated activation (c) was found for tracts that were part of the splenium. Negatively correlated activation (d) was found at the position that corresponded with the optic radiation.

Positively correlated active tracts (that is, active tracts that show a significant positive correlation with the task) found for a single subject during the tactile task and the visual task in the fDTI experiment are shown in Figure 2. (See Movie S1 and Movie S2 for these fDTI results in combination with conventional fMRI results.) The results of all individuals for the fDTI experiment were placed in a common space to study the accumulated activation patterns for each task separately (Figure 3). For the tactile task, positively correlated activation was most consistently found for the afferent tracts of the left sensory thalamocortical tract and the splenium of the corpus callosum (Figure 3a). Negatively correlated activation was most consistently found for the afferent tracts of the right sensory thalamocortical tract (Figure 3b). For the visual task, positively correlated activation was most consistently found for the genu and splenium of the corpus callosum and to a lesser extent for the tracts of the right sensory thalamocortical tract (Figure 3c). For the visual task, negatively correlated activation was most consistently found for both left and right optic radiations. The average percent FA-signal change, averaged over all active tracts of all subjects were 0.98% and −1.40%, for the tactile positive and negative correlating tracts, and 1.06%, and −1.45%, for the visual positive and negative correlating tracts, respectively. The results for the average percent signal change for the same set of active tracts computed for the radial diffusivity was: −1.49%, 1.91%, −1.38%, 1.34%. For the parallel diffusivity the average percent signal change was: 0.39%, −0.44%, 0.27%, −0.47% and for the mean diffusivity: −0.03%, 0.08%, −0.21%, 0.04%. Note that the average percent signal changes for radial diffusivity and parallel diffusivity have opposite signs leading to reduced signal changes in mean diffusivity as the radial diffusivity and parallel diffusivity changes partially cancel each other out.

**Figure 4:**
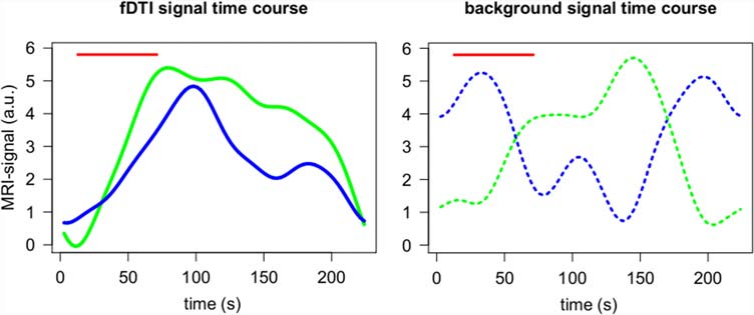
Time course experiment. The time courses of the diffusion weighted MRI signal for the visual and the tactile task measured perpendicular to the tract's main direction using a single stimulus. An increase in glial cell volume is expected to lead to an increase of the measured signal. The tactile and the visual stimulus period (red horizontal line) started at t = 12 s and ended at t = 72 s. a) The results represent the averages of 15 measurements (3 subjects; 5 measurements per subject) smoothed using a Gaussian kernel with sigma = 14 s. For the tactile stimulus (green) the maximum signal was found at t = 78 s. For the visual stimulus (blue) the maximum signal was found at t = 98 s. b) The averages of the signals of the background voxels that were used to correct for global signal variations (dashed blue line for the visual task and green dashed line for the tactile task). Note that these signals were independently scaled for visualization.

The results of the time course experiment showed that with the same stimulus length for the tactile task and the visual task the signal maximum was reached at 78 seconds and 98 seconds, respectively (Figure 4).

## Discussion

In this study we present a method to measure task-related FA changes hypothesized to reflect white matter activity in the human brain. We found expected activation patterns of white matter tracts for both the tactile and the visual task albeit that for the visual task the correlation between signal change and task appears to be reversed. During the tactile task the sensory thalamocortical tract was active while the optic radiation was active during the visual task. These white matter tracts were expected to become active based on their anatomical connections with the sensory and occipital cortices, which are gray matter areas known to be involved in tactile and visual tasks.

In the tactile task the majority of the subjects showed the expected tactile task-specific white matter activation patterns. Positively correlated activation was found for the left sensory thalamocortical tract. The negatively correlated activation that was found in the ipsilateral sensory thalamocortical tract may reflect a suppression of inputs from the opposite hand as reported earlier during fMRI experiments [33], [34].

In the visual task, the majority of subjects showed positively correlated active callosal tracts connecting homotopic visual regions [35] crossing the splenium as well as positively activated tracts in the frontal region. However, because similar activation patterns were found for the results of the tactile experiment we must consider the possibility that these activation patterns are not directly linked to the visual stimulus. Activation of the optic nerve was less likely to be detected because of known large susceptibility artifacts at the base of the brain. The most prominent activation, as expected, was along the optic radiation [36].

The majority of subjects showed negatively correlated activation in the optic radiation but deactivation of the optic radiation (Figure 3d) is less likely during a visual task. If, however, the underlying contrast mechanism does have a slow varying response function, as is suggested by the time course experiment, a more plausible explanation would be that this reversal of the sign of the activation actually reflects a difference in time course of the measured signal for the tactile and visual task. This could possibly be due to the checkerboard stimulus being perceived as a more intense stimulus than the tactile stimulus. Such an intensity difference could lead to differences in the time needed for the measured signal to reach its maximum or to return to baseline. Indeed the results of the time course experiment (Figure 4) suggest a difference in when the signal maximum is reached. For the tactile task the maximum signal is reached at 78 seconds, almost directly after the end of the stimulation period. The maximum signal for the visual task however is reached at 98 seconds. Thus for checkerboard stimulation experiments using stimulation and resting periods of 60 seconds, the signal maximum of a stimulation period is not reached within the stimulation period itself but well within the subsequent resting period. The substantial lag between the end of the checkerboard stimulus and the signal maximum could therefore result in an anti-correlation between the task and the measured fDTI signal resulting in negative activation. This because, for visual stimuli, the period of acquisition of the diffusion weighted volumes (b1000) (see Figure 1e) of the resting period is now positioned more closely in time to the (delayed) signal maximum (Figure 1e blue arrow) than the b1000 of the stimulus period itself.

Our fDTI method is based on the assumption that changes in FA are a sign of local fiber activity. At this point the biophysical underpinning of the measured fDTI signal is not known. Several different physiological processes that are part of (or accompany) fiber activity could, in theory, alter the diffusion profile and therefore the measured FA-value. For instance, a possible mechanism that could change the shape of the ECS and therefore the measured FA-value is activity-related swelling of glial cells [15]. As a result of neural fiber activity, the level of potassium (K^+^) in the ECS increases (for sensory stimulation up to 0.4 mM and for visual stimulation up to 1 mM [37], [38]) and leads to cell swelling of its surrounding glial cells (particularly oligodendrocytes and fibrous astrocytes). This glial cell swelling would then lead to anisotropic changes of the ECS in white matter [12], [13]. Such an anisotropic change of the ECS could result in a measurable increase in FA because DTI with a diffusion weighting and echo time as used in this study (b-factor = 1000 s/mm^2^, TE = 74 ms) is believed to be primarily sensitive to the diffusion of water in the ECS [39]. Indeed, changes in the diffusion profile due to changes of the ECS in white matter have been shown *in vitro* using diffusion weighted imaging in the rat optic nerve [15]. In that study a larger relative decrease in diffusivity was found in the radial direction than in the parallel direction as is reflected by an increased FA-value. Also it was shown that the time required for full glial cell swelling may involve tens of seconds or longer, depending on the strength of the stimulus [13], [40]. Intrinsic optical signal (IOS) measurements during electrical stimulation of the rat optic nerve [17] showed a response function that was in the order of tens of seconds and was interpreted as a decrease of ECS volume due to glial cell swelling. Interestingly, the measured IOS signal continuously increased during the stimulation period and reached its maximum well after the end of the stimulus period similar to the results of our time course experiment. This similarity in time course suggests that glial cell swelling could indeed be one of the underlying physiological mechanisms that is responsible for the measured FA-signal change. Considering their results a coarse estimate can be made indicating that an increase of the concentration of K^+^ in the ECS with 1 mM would lead to a decrease of the ECS in the order of 1%. But how this 1% decrease relates to changes in the measured FA-value is very difficult to determine because the relation between the measured diffusivity and ECS volume is not well understood [13], [41].

Recent findings indicate that elevated levels of K^+^ in the ECS not only result in volumetric changes but also result in complex morphological changes of astrocytes [18], [19], [41]–[43]. If glial cell swelling is the underlying mechanism of the signal changes measured in this study then an overall decrease of the measured diffusion profile (the mean diffusivity) would be expected. However, if morphological changes of glial cells underlie the measured signal changes then changes in the shape of the diffusion profile (reflected by the FA) would be more probable then changes in mean diffusivity. The results of the average percent FA-signal change and the average percent signal change in mean diffusivity do suggest that morphological glial cell changes are more likely to be responsible for the measured signal changes measured in this study then glial cell swelling because the changes in FA were larger than the changes in mean diffusivity.

In gray matter an activity-related increase in blood flow in the capillaries can be detected with intravoxel incoherent motion [3]. Although intravoxel incoherent motion typically uses moderate diffusion weighting (up to b = 700 s/mm^2^) it was suggested [28] that even with stronger diffusion gradients as used in this study (b = 1000 s/mm^2^) changes in blood flow in the smallest capillaries could still contribute to the measured MRI signal. If the capillaries in white matter have a preferential direction then the changes in blood flow within a voxel may be anisotropic an alter the shape of the diffusion profile, which would be reflected by a change in FA.

Another possible mechanism, closely linked to changes in blood flow, that could lead to changes in FA would be an activity-related increase in microvascular blood volume [22]. An increase in blood volume within a voxel would result in a decrease of the volume of the white matter's parenchyma in that voxel [22] hence reducing the fraction of tissue that is responsible for the measured anisotropy with DTI. In addition, the dilatation of the blood vessels may result in an anisotropic shape change of the extra-cellular space (ECS).

Changes in the level of blood oxygenation resulting in changes in local susceptibility (which is the contrast mechanism of BOLD-fMRI) could also alter the FA-value. If the microvascular system in white matter has a preferential direction within a voxel then anisotropic task-related changes in susceptibility could potentially contribute to the measured FA-signal change, as was shown in a computer simulation [23]. However, the effect of possible task-related anisotropic susceptibility changes on the FA measurements is probably limited. If the capillary bed in white matter has a preferential direction, then task-related changes in susceptibility will be very small in all tracts for which the preferential direction of the capillary bed runs parallel to the main magnetic field. This because a change in the level of blood oxygenation in capillaries running parallel to the main magnetic field does not lead to changes in susceptibility [30]. But the results of the fDTI experiments showed active tracts in all major directions. If task-related anisotropic susceptibility changes do substantially contribute to the measured FA-signal changes then one would expect that no active tracts were found in at least one of the major directions.

The last three possible mechanisms are all based on microvascular changes. Using a two-compartment model [28] we estimated that in the fDTI experiment the maximum activity-related signal changes by blood volume changes is 0.5% for radial diffusivity and for parallel diffusivity −0.72%. These results show that for parallel diffusivity the task-related signal changes could, in theory, be explained by activity-related blood volume changes. However, for radial diffusivity the maximum possible task-related signal change induced by the changes in blood volume are about three times smaller than the measured average percent FA-signal change. This suggests that the measured signal changes cannot be explained by microvascular changes alone and that other contrast mechanisms are responsible for the major part of the measured signal change.

The order of the acquisition of the different diffusion weighted volumes of a single DTI scan may have an effect on the measurements due to the slow varying time course of the measured signal. Although the ordering itself could not be responsible for the measured FA-signal change (as the ordering of the different diffusion directions is identical for the DTI scans collected during rest as for the DTI scans collected during stimulation) it may introduce different sensitivities in different directions. In future experiments the influence of such possible order-specific effects could be reduced by using a round robin scheme for the diffusion gradients of consecutive DTI scans.

The sheer presence of a person at the entrance of the MRI bore (e.g. to apply the tactile stimulus) can lead to changes in the experienced main magnetic field strength within the scanner's field of view. However, it is not likely that these changes influence the measurements because the FA is a relative measure and these changes do not occur within a single fDTI acquisition.

Although the results of the time course experiment suggest the need for different timing parameters for different types of single stimulus intervals, it does not provide information on possible saturation effects in the case when repetitive stimulation periods are used. For glial cell swelling for example, saturation effects may be due to a resting period that is too short to allow the glial cells to shrink to their normal size. Additional experiments are needed to study the fDTI signal during repetitive stimulation in order to optimize the experimental design.

The results of the fDTI method are to a large extent determined by the quality of the conventional DTI scan and the applied fiber tracking algorithm. For instance, it is known that sensory information enters the thalamus via the ventral spinothalamic fasciculus and is then projected via the thalamus onto the sensory cortex via the sensory thalamocortical tract. However, results for the tactile task show some active tracts that appear to start in the thalamus and run into the sensory cortex as expected, while other tracts appear to originate in the pons, passing the thalamus and run directly towards the sensory cortex. The latter tracts could be the result of the fiber tracking algorithm erroneously combining separate fibers (in this case tracts from the spinothalamic fasciculus and sensory thalamocortical tract) into one single tract. Besides erroneously combining separate tracts, the erroneously splitting of one tract into parts also occurs. For instance, fiber tracking algorithms utilising the single tensor model cannot adequately reconstruct crossing fibers [7]. Even if all constituent (separate) parts of a set of crossing active fibers were successfully reconstructed, these parts may be too short and therefore insufficiently profit from the increased sensitivity of the tract based statistics approach to be marked as active. The number of false negatives of the fDTI method is therefore directly related to the quality of the fiber tracking algorithm used.

One of the limitations of this study is the relatively large voxel size used (64 mm^3^ in the fDTI experiment) which leads to considerable partial voluming. This limits the detection of tract activation to the major tracts such as the optic radiation and the sensory thalamocortical tract. The latter, for instance, has a diameter of several millimeters [44], [45].

**Figure 5:**
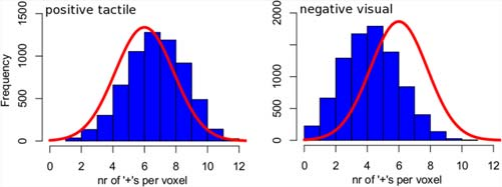
Histogram distributions of voxels that are part of active fibers. Here two histograms are shown of the number of ‘+’s per voxel, for all voxels along active fiber tracts, separately for positive correlation and negative correlation, combined for all subjects. In the case that there would not be an activity-related signal change, the distribution of +'s in these voxels would follow a normal distribution (solid red line). If only a small group of voxels (the active voxels) is responsible for a tract to be considered active then these voxels must show a high correlation (i.e. a high number of +'s) with the task while the distribution of +'s of the other (non-active) voxels that are part of the active tract would remain unchanged. This would result in a histogram built up from a large and a small normal distribution. The large group of non-active voxels would produce the original (large) normal distribution (solid red line) while the small group of active voxels would produce a second smaller distribution left (negative correlation) or right (positive correlation) from the original distribution. However, if not a small group but the majority of the voxels along the active fibers belong to the set of active voxels, one would expect a single normal distribution which is shifted to left (negative correlation) or to the right (positive correlation). Left, the histogram for the (positive) active voxels for the tactile task is shown while on the right side the histogram for the (negative) active voxels of the visual task is shown. Both histograms appear to follow a single normal distribution but are shifted to the right (positive correlation with tactile task) or to the left (negative correlation with visual task). This suggests that the task-related signal changes found with fDTI indeed occur along large parts of the fibers and are not confined to small parts of the fibers.

The sign-test, the statistical test used in this study to test for tract activation, is a non-parametric statistical test that ignores the size of the measured differences between active and rest conditions and only takes the signs of these differences into account. This is an important feature of the sign-test as the FA-value along a fiber tract will vary considerably and the sizes of the task-related FA signal changes at different levels of FA-values are incomparable. An important aspect of tract-based analysis is that all direct comparisons of FA signals (when the FA-signal changes are encoded into a series of ‘+’s and ‘−’s) are done within a voxel where the FA signals for task and rest conditions will be of similar magnitude. As the sign-test limits the effect of possible outliers it is very robust in the sense that tracts will not be considered active on the basis of the FA signal behavior of a few voxels alone (See Figure 5). This reduces the chance of finding spurious active tracts, which is important because the aim of this study was to show that the fDTI method can successfully be applied to detect activation of white matter tracts. Moreover, we applied the conservative Bonferroni correction for multiple testing thereby assuming that each test for fiber activation is an independent test. However, this is not the case as fibers may (partly) overlap and as a consequence the Bonferroni correction is too conservative. On the other hand the degrees of freedom for the sign-test are based on the assumption that the signals of neighbouring voxels are independent and a violation of this assumption may lead to an overestimation of fiber activation. Further research is needed to obtain a better estimate of the required correction factor for multiple testing.

**Figure 6:**
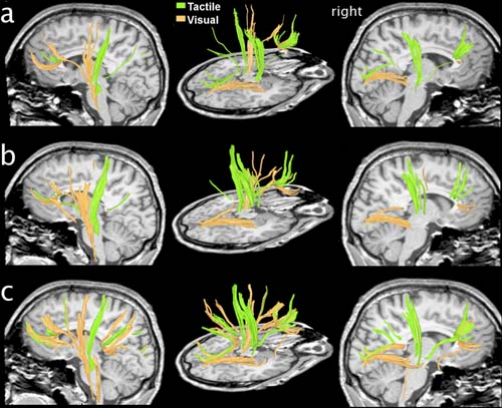
Positively correlated fDTI results for subject #5 computed in three different ways. (a) The original active tracts found based on changes in FA using the sign-test (See Figure 1). (b) Analysis of the same data set using conventional t-statistics as in ROI/VOI based fMRI. For each voxel in the fDTI scan a multiple regression was carried out. Two regressors were used, one encoding for the stimulus (0/1) and the other representing possible linear scanner drift. Each tract that was tested for activation was considered active if the mean of the stimulus regressor values of the voxels in the tract was found significantly higher than zero using a one-sided Student's t-test (p<0.05; Bonferroni corrected for the number of tested tracts). (c) Active tracts based on changes in the radial diffusion component using the sign-test. The three different methods yielded very similar activation patterns. The similar results for (a) and (b) indicate that the results do not depend on the chosen statistical method. The similar results for (a) and (c) indicate that the measured signal change stems predominantly from a change in diffusion in the radial direction.

To make sure that these findings do not depend on the choice of a particular statistical analysis method used the data was also analyzed using parametric statistics. In this analysis conventional t-statistics as in ROI/VOI based fMRI (here a VOI was defined by the voxels that were part of the tract that was tested for activation) were used. First, t-tests are used to test differences in mean FA value between active and rest conditions *per voxel*. Second, a t-test is used to test whether the average t-value of all voxels in the VOI (i.e. the tract to be tested for activation) is significantly greater than-or smaller than zero. In that case the tract is considered to show a positive correlation or negative correlation, respectively. Note, that in this particular case we conduct a t-statistic over t-values and not a t-statistic over b-values because at this stage we only want to establish tract activation. The results of this analysis did not change our findings. In Figure 6 the results of the analyses based on the sign test as well as on parametric statistics are shown for a single subject.

It was shown that the FA measure is robust against noise induced bias for higher values (FA>0.4) but it is not rotationally invariant when 6 different diffusion gradients are used (here used in fDTI data acquisition) [46]. Possible effects of this rotational dependency on the fDTI results are expected to be very limited because deviations due to this dependency form a constant factor within a voxel over the subsequent rest and task conditions (assuming no gross subject motion).

In conclusion, we propose a non-invasive method to identify white matter tract activation *in-vivo*. Active tracts were identified using a tract-based statistical analysis. The results of the experiments indicate that the task-related FA signal changes can be detected but have a low temporal resolution (tens of seconds), which is in line with previously reported results of glial cell swelling in white matter. Furthermore the results suggest that there may be a relation between the time the signal reaches its maximum and the intensity of the presented stimuli. The low temporal resolution as well as the possible dependency on stimulus intensity should be to taken into account in the design of future experiments. We believe that the fDTI method may become a valuable tool to study the brain's active connections that could help us to get a better understanding of the functional architecture of the neural networks in the human brain.

## Supporting Information

Movie S1 fDTI data combined with fMRI data for the tactile stimulus (subject #5). The left and right thalamus were automatically segmented using the anatomy scan and are shown in dark green and dark red respectively.
(5.27 MB MPG)

Movie S2 fDTI data combined with fMRI data for the visual stimulus (subject #5). The left and right thalamus were automatically segmented using the anatomy scan and are shown in dark green and dark red respectively.
(4.92 MB MPG)
